# Health Care Engagement in Disease Prevention and Management: Factors Influencing Chronic Disease Program Referral Adherence Among Non-Hispanic Black and Hispanic Men With Chronic Conditions

**DOI:** 10.1177/15579883241288978

**Published:** 2024-10-24

**Authors:** Caroline D. Bergeron, Cynthia L. Cisneros Franco, Ledric D. Sherman, Kristin Pullyblank, Wendy Brunner, Arica A. Brandford, Chung Lin Kew, Matthew Lee Smith

**Affiliations:** 1 LIFE Research Institute, University of Ottawa, Ottawa, ON, Canada; 2 Department of Sociology, College of Arts and Sciences, Texas A&M University, College Station, TX, USA; 3 Department of Health Behavior, School of Public Health, Texas A&M University, College Station, TX, USA; 4 Center for Health Equity and Evaluation Research, Texas A&M University, College Station, TX, USA; 5 Bassett Research Institute, Bassett Healthcare Network, Cooperstown, NY, USA; 6 Houston Methodist Research Institute, Houston, TX, USA; 7 Center for Community Health and Aging, Texas A&M University, College Station, TX, USA

**Keywords:** evidence-based programs, disease self-management, health care provider referrals, non-Hispanic Black and Hispanic men, chronic conditions

## Abstract

This study aimed to identify factors associated with being referred to an evidence-based disease prevention and management program by a health care provider and adherence to such referrals by non-Hispanic Black and Hispanic men. Utilizing a cross-sectional design, data were collected via an internet-based questionnaire from a national sample of 1,679 non-Hispanic Black and Hispanic men ages 40 years and older with one or more chronic diseases. A 105-item survey assessed program referral and attendance, chronic conditions and medications, disease symptoms, support, communication during physician visit, health care frustrations, disease self-management efficacy, barriers to self-care, helpfulness of learning from others for self-care, and sociodemographics. Binary logistic regression models were fitted to assess factors associated with referrals to a disease prevention and management program and attendance. Results indicated that approximately 23% of participants were referred to a program, and 19.2% reported attendance. Factors associated with being referred to and attending a program included being younger, having more chronic conditions, taking more medications daily, having higher pain scores, reporting more health care frustrations, and reporting better communication with physicians during visits. Men referred to attend a chronic disease program by a health care provider were 16.86 times more likely to attend a chronic disease program (*p* < .001). These findings suggest the importance of health care engagement for non-clinical disease prevention and management programs, particularly among non-Hispanic Black and Hispanic men with complex disease profiles.

## Introduction

Evidence-based programs (EBPs), such as Chronic Disease Self-Management Education programs (Smith, Towne, et al., 2017), are health promotion interventions that can help adults prevent and manage their chronic diseases (National Council on Aging, 2021). By focusing on improving health behaviors, managing disease symptomatology, and reducing disease progression (Ory, Ahn, Towne, et al., 2015), EBPs complement the clinical care provided by the health care system (Ahn et al., 2013). Important community-clinical linkages are made, where health care entities collaborate with community-based organizations implementing the programs to engage patients in clinical care or to raise program awareness and refer patients to EBPs (Faro et al., 2021).

The effectiveness of EBPs for disease management has been well documented over the past few decades (Ahn et al., 2013; Ory, Ahn, Jiang, Lorig, et al., 2013; Ory, Ahn, Jiang, Smith, et al., 2013). EBPs have been found to improve health care outcomes (e.g., improved hemoglobin levels, reduced disease symptomatology; Ory, Ahn, Jiang, Smith, et al., 2013) and quality of life (Peytremann-Bridevaux et al., 2008), decrease health services utilization (e.g., emergency room visits, hospitalizations; Hisashige, 2012; Ory, Ahn, Jiang, Smith, et al., 2013), and reduce health care costs (Hisashige, 2012).

Men often underutilize preventive and regular health care services, including EBPs (Howell et al., 2022). In the United States, only about 20% of EBP participants are men (Mingo et al., 2014), with non-Hispanic Black and Hispanic men representing an even smaller proportion (Smith et al., 2022). Factors may contribute to the underrepresentation of non-Hispanic Black and Hispanic men in EBPs, including the role of masculinity (Chan & Corvin, 2015), fears and stress associated with self-management (Hawkins, 2019), self-efficacy (Mansyur et al., 2023), costs (Hawkins, 2019), and lack of transportation (Ferretti & McCallion, 2019).

Health care providers, such as physicians and nurses, play a vital role in implementing EBPs effectively. They directly interact with patients living with chronic conditions, influencing decision-making and empowering patients in their self-management efforts (Petersson et al., 2022). In the shared decision-making for planning and working toward health goals, health care professionals need to be aware of the programs and resources accessible to their patients in the community, assess the potential benefits of participating, and make the appropriate referrals to EBPs (Alsayed Hassan et al., 2020).

Evidence-based research remains insufficient regarding the proportion of health care referrals that result in program attendance, especially among racially and ethnically diverse male populations that less frequently attend EBPs. The purposes of this study were to identify factors associated with being referred to a disease prevention/management program by a health care provider, as well as the adherence to such referrals, among non-Hispanic Black and Hispanic men ages 40 years and older with one or more chronic conditions.

## Materials and Methods

### Design and Sample

Cross-sectional data were collected using an internet-based questionnaire between September and November 2019. Participants were recruited nationally from a Qualtrics panel as part of a larger study investigating health-related attitudes and behaviors, and facilitators to and barriers of medical and preventive health service utilization, among non-Hispanic Black and Hispanic men ages 40 years and older with one or more chronic diseases (Smith et al., 2022). Participants completed a 105-item survey developed by the research team, which largely comprised content from other validated sources (The Atlantic Philanthropies, 2009; Ory, Ahn, Jiang, Lorig, et al., 2013; Ory, Ahn, Jiang, Smith, et al., 2013).

Overall, 2,028 men who met the inclusion criteria completed the questionnaire. Given this study’s focus on men being referred by a health care professional to attend a chronic disease prevention/management program, men who did not report a doctor visit in the past year were omitted from analyses (*n* = 349). The remaining analytic sample consisted of 1,679 non-Hispanic Black and Hispanic men ages 40 years and older with one or more chronic conditions. This study was reviewed and approved by the Institutional Review Board at Texas A&M University (#2018-1684).

### Measures

#### Dependent Variables

Two dependent variables related to chronic disease programming were included in this study. First, participants were asked, “In the past year, has your health provider referred you to a program to help you prevent or manage a chronic illness?” Second, participants were asked, “In the past year, have you attended a program to help you prevent or manage a chronic illness?” Responses to both items were no (scored 0) or yes (scored 1) and used binarily in logistic regression analyses (no served as the reference category in both regression models).

#### Sociodemographics

Sociodemographic measures included age (range 40–89 years), race/ethnicity (non-Hispanic Black, Hispanic), educational attainment (high school or less, some college/2-year degree, 4-year degree, or more), and marital status (married/partnered, never married, divorced/separated, widowed).

#### Chronic Conditions and Medications

Chronic conditions were measured using a self-reported “check all that apply” list that presented 19 chronic physical and mental health conditions, such as asthma, arthritis, cancer, chronic pain, diabetes, depression or anxiety, and heart disease (range 0–19). In addition, participants reported the number of different medications taken daily (range 0 to ≥ 6).

#### Disease Symptoms

Participants rated their problems with fatigue and pain using a scale from best possible (scored 0) to major problem (scored 10; Ory, Ahn, Jiang, Lorig, et al., 2013; Ory, Ahn, Jiang, Smith, et al., 2013).

#### Support

The frequency of participants receiving the help and support needed to improve their health and manage health problems was measured using a single item with a 5-point scale ranging from never (scored 1) to always (scored 5; The Atlantic Philanthropies, 2009; Smith et al., 2013, 2022).

#### Communication During Physician Visit Index

This index comprised four questions related to interactions with doctors during visits (Ory, Ahn, Jiang, Lorig, et al., 2013; Ory, Ahn, Jiang, Smith, et al., 2013; Smith et al., 2022). Examples of items included the participant asking questions about things they want to know and things they do not understand about their treatment, or discussing personal problems that may be related to their illness. Each item was scored using a 5-point scale with response options of never (scored 1), almost never, sometimes, fairly often, and always (scored 5). Responses were summed to create a composite score ranging from 4 to 20, with higher scores translating to better communication by the participants. The Cronbach’s alpha for this index in the current sample was .791.

#### Health Care Frustrations Index

This index comprised six items that assessed whether participants felt frustrations during health care interactions. Examples of items included the participants feeling tired of describing their same conditions and problems every time they go to a hospital or doctor’s office or wishing their doctor had more time to spend talking with them. Each item was scored using a 3-point scale with response options of never (scored 1), occasionally, and frequently (scored 3). Responses were summed to create a composite score ranging from 6 to 18, with higher scores translating to higher health care frustrations (Smith et al., 2022). The Cronbach’s alpha for this index in the current sample was .860.

#### Disease Self-Management Efficacy Index

This index comprised 10 items that participants agreed or disagreed with using a 4-point scale (Ory, Ahn, Jiang, Lorig, et al., 2013; Ory, Ahn, Jiang, Smith, et al., 2013; Smith et al., 2022). Examples of items included participants reporting their confidence about whether they could follow through on medical treatments they may need to do at home or maintain lifestyle changes, like eating right or exercising. Response choices for these 10 items were strongly disagree (scored 1), disagree, agree, and strongly agree (scored 4). Responses were summed to create a composite score ranging from 10 to 40, with higher scores translating to higher efficacy. The Cronbach’s alpha for this index in the current sample was .887.

#### Barriers to Self-Care Index

This index comprised five items related to their barriers to managing their health conditions (The Atlantic Philanthropies, 2009; Smith et al., 2022). Examples of items included the extent to which participants needed help learning what they should be doing to take better care of their health or needed help learning how to take better care of their health in a way that works for them and their life. Each item was scored on a 4-point scale with response options of strongly disagree (scored 1), disagree, agree, and strongly agree (scored 4). Responses were summed to create a composite score ranging from 5 to 20, with higher scores translating to more barriers. The Cronbach’s alpha for this index in the current sample was .845.

#### Helpfulness of Learning From Others for Self-Care Index

This index comprised four items related to how helpful learning from others was to their own self-care (The Atlantic Philanthropies, 2009; Smith et al., 2013). Examples of items included the extent to which participants found it helpful to get practical tips and advice from other people who have health problems or set goals and work together with other people who are trying to improve their health. Each item was scored on a 4-point scale with response options of not at all helpful (scored 1), not too helpful, somewhat helpful, very helpful (scored 4). Responses were summarized to create a composite score ranging from 4 to 16, with higher scores translating to perceiving that learning from others is more helpful. The Cronbach’s alpha for this index in the current sample was .838.

### Analysis

All analyses were performed using SPSS version 28 (IBM Corp., 2021). Descriptive statistics were calculated for all variables of interest. Bivariate analyses were performed to compare sample characteristics by each dependent variable (i.e., referred to chronic disease program, attended a chronic disease program). Pearson’s chi-square tests were used to assess differences for categorical variables, and independent sample *t* tests were used to assess mean differences for continuous variables. Two binary logistic regression models were applied to assess factors associated with program referral and attendance, respectively (i.e., not being referred or not attending served as the reference category in each respective model). Then, to assess the direct association of program referral on attendance, a third binary logistic regression model was fitted to identify factors associated with attending a chronic disease program (i.e., with being referred as a primary predictor variable in the model). Results with *p* values under .05 were deemed statistically significant for all analyses in this study.

## Results

Table 1 provides sample characteristics by program referral and program attendance variables. Among the 1,679 men in the sample, 23.4% (*n* = 393) were referred by a health care professional to attend a chronic disease program and 19.2% (*n* = 323) reported attending a chronic disease program. Of the 23.4% of men referred to attend a chronic disease program, 60.3% (*n* = 237) attended a chronic disease program. Approximately, 60% of participants self-identified as non-Hispanic Black and 40.3% self-identified as Hispanic. On average, participants were age 57.33 (± 10.00) years, had 4.08 (± 2.97) chronic conditions, and took 3.65 (± 1.92) medications daily. About 20% of participants had a high school education or less, 42.5% attended some college or had a 2-year degree, and 37.8% had a 4-year college degree or more. Over half of participants (54.0%) were married or partnered, 23.7% were never married, 18.1% were divorced or separated, and 4.2% were widowed.

**Table 1.** Sample Characteristics by Program Referral and Attendance

	Total (*n* = 1,679)	Referred to program				Attended program			
Variables		No (*n* = 1,286)	Yes (*n* = 393)	χ^2^ or *t*	*p*	No (*n* = 1,356)	Yes (*n* = 323)	χ^2^ or *t*	*p*
Health care provider referred to disease program				—	—			556.98	< .001
No	76.6%	—	—			88.5%	26.6%		
Yes	23.4%	—	—			11.5%	73.4%		
Age (40–89)	57.33 (± 10.00)	57.92 (± 9.79)	55.42 (± 10.43)	4.36	< .001	57.80 (± 9.90)	55.35 (± 10.15)	3.98	< .001
Race/ethnicity				0.77	.380			2.39	.122
Non-Hispanic Black	59.7%	59.1%	61.6%			58.8%	63.5%		
Hispanic	40.3%	40.9%	38.4%			41.2%	36.5%		
Education				0.47	.790			1.29	.525
High school or less	19.8%	20.1%	18.6%			20.3%	17.6%		
Some college or 2-year degree	42.5%	42.2%	43.3%			42.4%	42.7%		
4-year degree or more	37.8%	37.6%	38.2%			37.3%	39.6%		
Marital status				2.57	.462			1.00	.801
Married or partnered	54.0%	53.4%	55.7%			54.1%	53.3%		
Never married	23.7%	23.5%	24.4%			23.4%	25.1%		
Divorced or separated	18.1%	18.5%	16.8%			18.1%	18.3%		
Widowed	4.2%	4.6%	3.1%			4.4%	3.4%		
Number of chronic conditions (0–19)	4.08 (± 2.97)	3.86 (± 2.75)	4.80 (± 3.49)	−4.92	< .001	3.86 (± 2.80)	4.99 (± 3.43)	−5.51	< .001
Number of medications taken daily (0–6+)	3.65 (± 1.92)	3.56 (± 1.93)	3.96 (± 1.85)	−3.67	< .001	3.57 (± 1.93)	4.01 (± 1.84)	−3.74	< .001
Disease symptoms									
Fatigue (0–10)	3.45 (± 3.29)	3.18 (± 3.22)	4.36 (± 3.33)	−6.31	< .001	3.22 (± 3.24)	4.45 (± 3.31)	−6.14	< .001
Pain (0–10)	4.09 (± 3.36)	3.73 (± 3.25)	5.26 (± 3.43)	−8.08	< .001	3.81 (± 3.30)	5.22 (± 3.36)	−6.86	< .001
Get the help/support to improve health and manage health problems (1–5)	3.76 (± 1.04)	3.76 (± 1.04)	3.76 (± 1.04)	−0.04	.968	3.75 (± 1.04)	3.79 (± 1.05)	−0.69	.488
Scales for health-related perceptions									
Communication During Physician Visit Index (4–20)	14.47 (± 3.36)	14.19 (± 3.36)	15.36 (± 3.19)	−6.08	< .001	14.21 (± 3.36)	15.55 (± 3.13)	−6.54	< .001
Health Care Frustrations Scale (6–18)	9.31 (± 3.11)	9.02 (± 2.94)	10.27 (± 3.43)	−6.48	< .001	9.06 (± 2.97)	10.38 (± 3.43)	−6.35	< .001
Disease Self-Management Efficacy Scale (10–40)	33.16 (± 4.94)	33.11 (± 4.99)	33.32 (± 4.78)	−0.72	.473	33.06 (± 4.96)	33.57 (± 4.83)	−1.66	.096
Barriers to Self-Care Scale (5–20)	11.31 (± 3.65)	10.96 (± 3.50)	12.46 (± 3.90)	−6.82	< .001	11.00 (± 3.58)	12.63 (± 3.68)	−7.32	< .001
Helpfulness of Learning from Others for Self-Care Scale (4–16)	11.80 (± 2.88)	11.63 (± 2.92)	12.37 (± 2.68)	−4.53	< .001	11.62 (± 2.96)	12.57 (± 2.39)	−6.10	< .001

Similarities were observed when comparing sample characteristics for participants who were referred to a program and participants who attended a program. On average, participants who were referred to a program (*t* = 4.36, *p* < .001) or attended a program (*t* = 3.98, *p* < .001) were younger, had more chronic conditions (*t* = −4.92, *p* < .001 and *t* = –5.51, *p* < .001, respectively), and took more medications daily (*t* = –3.67, *p* < .001 and *t* = –3.74, *p* < .001, respectively). On average, participants who were referred to a program or attended a program reported greater fatigue (*t* = –6.31, *p* < .001 and *t* = –6.14, *p* < .001, respectively) and pain (*t* = –8.08, *p* < .001 and *t* = –6.86, *p* < .001, respectively). Furthermore, on average, participants who were referred to a program or attended a program reported better communication during physician visits (*t* = –6.08, *p* < .001 and *t* = –6.54, *p* < .001, respectively), more health care frustrations (*t* = –6.48, *p* < .001 and *t* = –6.35, *p* < .001, respectively), more barriers to self-care (*t* = –6.82, *p* < .001 and *t* = –7.32, *p* < .001, respectively), and believed it to be more helpful to learn from others about self-care (*t* = −4.53, *p* < .001 and *t* = –6.01, *p* < .001, respectively). A significantly larger proportion of participants who were referred by a health care provider attended a chronic disease program (χ^2^ = 556.98, *p* < .001).

Table 2 presents the results for logistic regression analyses. For each additional year of age, men were less likely to be referred by a health care provider to attend a chronic disease program (odds ratio [OR] = 0.98, *p* = .020). Each additional chronic condition (OR = 1.07, *p* = .001) and medication taken daily (OR = 1.10, *p* = .011) increased the odds of men being referred to attend a chronic disease program by a health care provider. For each unit increase in pain problems (OR = 1.09, *p* < .001), better communication during physician visits (OR = 1.08, *p* < .001), health care frustrations (OR = 1.06, *p* = .015), disease self-management efficacy (OR = 1.03, *p* = .047), and barriers to self-care (OR = 1.08, *p* < .001), the odds of being referred by a health care provider to attend a chronic disease program increased.

**Table 2.** Binary Logistic Regression Models

	Referred to program				Attended program				Attended program (including referral variable)			
	OR	*p*	95% CI		OR	*p*	95% CI		OR	*p*	95% CI	
Variables			Lower	Upper			Lower	Upper			Lower	Upper
Age	0.98	.020	0.97	1.00	0.98	.039	0.97	1.00	0.99	.406	0.97	1.01
Non-Hispanic Black	1.00	—	—	—	1.00	—	—	—	1.00	—	—	—
Hispanic	0.82	.120	0.64	1.05	0.74	.035	0.56	0.98	0.77	.114	0.55	1.07
Education: high school or less	1.00	—	—	—	1.00	—	—	—	1.00	—	—	—
Education: some college or 2-year degree	1.10	.555	0.80	1.54	1.14	.486	0.79	1.63	1.16	.510	0.75	1.78
Education: 4-year degree or more	1.13	.474	0.80	1.60	1.29	.179	0.89	1.88	1.34	.201	0.86	2.09
Marital status: married	1.00	—	—	—	1.00	—	—	—	1.00	—	—	—
Marital status: never married	0.75	.065	0.55	1.02	0.81	.207	0.58	1.13	0.94	.753	0.63	1.40
Marital status: divorced/separated	0.83	.278	0.60	1.16	1.02	.916	0.72	1.45	1.22	.354	0.80	1.85
Marital status: widowed	0.65	.203	0.33	1.27	0.76	.454	0.38	1.55	0.98	.966	0.44	2.19
Number of chronic conditions (range 0–19)	1.07	.001	1.03	1.11	1.09	< .001	1.05	1.14	1.07	.007	1.02	1.13
Number of medications taken daily (0–6+)	1.10	.011	1.02	1.18	1.11	.010	1.02	1.19	1.07	.131	0.98	1.17
Fatigue problem (0–10)	0.99	.705	0.95	1.04	1.01	.841	0.96	1.06	1.02	.473	0.96	1.09
Pain problem (0–10)	1.09	< .001	1.04	1.13	1.06	.023	1.01	1.11	1.01	.765	0.95	1.07
Frequency of getting the help/support needed (1–5)	1.11	.128	0.97	1.27	1.16	.056	1.00	1.34	1.11	.239	0.93	1.32
Communication During Physician Visit Index (4–20)	1.08	< .001	1.04	1.13	1.09	< .001	1.04	1.14	1.06	.041	1.00	1.12
Healthcare Frustrations Scale (6–18)	1.06	.015	1.01	1.11	1.07	.010	1.02	1.12	1.05	.129	0.99	1.11
Disease Self-Management Efficacy Scale (10–40)	1.03	.047	1.00	1.06	1.04	.009	1.01	1.08	1.04	.060	1.00	1.08
Barriers to Self-Care Scale (5–20)	1.08	< .001	1.04	1.13	1.10	< .001	1.05	1.15	1.07	.012	1.02	1.13
Helpfulness of Learning from Others for Self-Care Scale (4–16)	1.03	.180	0.99	1.08	1.05	.048	1.00	1.11	1.04	.166	0.98	1.11
Health provider referred to program: no	—	—	—	—	—	—	—	—	1.00	—	—	—
Health provider referred to program: yes	—	—	—	—	—	—	—	—	16.86	< .001	12.37	22.97
	Nagelkerke’s *R* square = 0.142				Nagelkerke’s *R* square = 0.159				Nagelkerke’s *R* square = 0.443			
	Referent category: not referred to program				Referent category: not attended program				Referent category: not attended program			

Regarding program attendance, for each additional year of age, men were less likely to attend a chronic disease program (OR = 0.98, *p* = .039). Relative to non-Hispanic Black participants, Hispanic men were less likely to attend a chronic disease program (OR = 0.74, *p* = .035). Each additional chronic condition (OR = 1.09, *p* < .001) and medication taken daily (OR = 1.11, *p* = .010) increased the odds of men attending a chronic disease program. For each unit increase in pain problems (OR = 1.06, *p* = .023), better communication during physician visits (OR = 1.09, *p* < .001), health care frustrations (OR = 1.07, *p* = .010), disease self-management efficacy (OR = 1.04, *p* = .009), and barriers to self-care (OR = 1.10, *p* < .001), the odds of attending a chronic disease program increased. For each unit increase in perceived helpfulness of learning from others for self-care, the odds of attending a chronic disease program increased (OR = 1.05, *p* = .048).

Regarding program attendance when accounting for a health care provider referral, men who were referred to attend a chronic disease program by a health care provider were 16.86 times more likely to attend a chronic disease program (*p* < .001). Each additional chronic condition increased the odds of men attending a chronic disease program (OR = 1.07, *p* = .007). For each unit increase in better communication during physician visits (OR = 1.06, *p* = .041) and barriers to self-care (OR = 1.07, *p* = .012), the odds of attending a chronic disease program increased.

## Discussion

The present study examined the experiences of non-Hispanic Black and Hispanic men living with one or more chronic conditions, focusing on factors influencing their access to chronic disease programs. Study results suggest that only 23% of all participants were referred to attend a chronic disease program by their health care provider, suggesting that referrals are scarce. Nevertheless, among those who received a referral, 60% attended a program. Previous studies have reported similar findings regarding limited provider referrals, yet positive enrollment to EBPs when a referral was made (Ali et al., 2019). As providers are considered a credible source of health information (Jackson et al., 2019), this finding suggests that provider referrals serve as effective nudges to seek and obtain additional support for chronic disease management.

Results by referral and attendance status merit further attention. Study participants who were younger, had more chronic conditions, took more medications daily, had greater issues with pain, reported better communication with their health care provider, had more health care frustrations, self-managed their disease more efficaciously, and had more barriers to self-care, were more likely to be referred to a chronic disease program. Younger men may receive more referrals potentially due to health care providers’ perceptions that their disease prevention and management are more urgent and their participation in chronic disease programs is more beneficial compared with older individuals (Ory et al., 2014), which could also indicate the presence of some age discrimination (Wyman et al., 2018).

Compared with older men, younger men may be more active and engaged with their providers, due in part to higher levels of literacy (Kobayashi et al., 2016), leading to better communication and greater referrals (Cooper & Roter, 2003). Effective patient–provider interactions during medical visits may help providers better understand the need for additional support for disease prevention and management (Cheng et al., 2023; Dineen-Griffin et al., 2019; Tavakoly Sany et al., 2020). Providers may recognize their patients’ health care frustrations and refer them to EBPs as a solution to addressing these barriers (Smith, Bergeron et al., 2017).

Additional efforts are needed to improve referrals to chronic disease programs. Increasing providers’ awareness about existing chronic disease prevention and treatment programs, making resources available to providers to facilitate referrals (e.g., referral procedures and marketing materials), and improving collaborations between clinical practice and community-based interventions could be important first steps (Faro et al., 2021; Pullyblank et al., 2022). Facilitating the referral process, for example, through electronic referral systems or automatic referrals, could also help improve referral and participation rates in chronic disease programs (Faro et al., 2021).

Participants who were referred to a chronic disease program and had more chronic conditions, reported better communication with their health care provider, and faced more barriers to self-care demonstrated a higher likelihood of attending a chronic disease program. Prior research has shown that individuals with a greater number of chronic conditions were more likely to report difficulties self-managing their conditions (Smith, Bergeron et al., 2017). Given that most chronic disease programs aim to facilitate self-management of chronic diseases (Ory, Smith, et al., 2013), the association between these specific factors and program adherence appears logical.

Those who reported better communication with their health care provider may have benefited from receiving more information and asking more questions about chronic disease prevention and management in general and participation in chronic disease programs (Brenk-Franz et al., 2017; Peimani et al., 2020). Although little information is available regarding how often patients and providers interacted or the extent of their relationships, open communication might have fostered a trusting and high-quality connection. This could result in reduced anxiety and enhanced self-efficacy for patients considering to participate in a chronic disease program to manage their conditions (Brenk-Franz et al., 2017; Peimani et al., 2020). This finding highlights the importance of educating patients and health care providers on effective communication about chronic disease prevention and management, especially on referral and engagement in chronic disease programs, ultimately contributing to enhanced patient outcomes and empowerment in their chronic disease self-management.

Further work is needed and essential to increase attendance in disease prevention and management programs among non-Hispanic Black and Hispanic men with chronic conditions. Several options are available to help promote the reach of chronic disease programs and potentially reduce barriers to participation, including virtual or online delivery (Lightfoot et al., 2022; McKeon et al., 2022), using mobile text messaging (Jones et al., 2014), and using self-directed materials and small group telephone support (Sheth et al., 2021), among others. Health care providers could also connect their patients to health coaches, community health workers, or other community navigators, who could explain the benefits and potential drawbacks of participating in chronic disease programs, motivate patients to enroll in the program, and provide ongoing support to ensure accountability (Gordon et al., 2015).

The following limitations should be taken into account when interpreting the study results. This study used a cross-sectional design, and therefore causality and temporality between the reported factors and program referral and attendance cannot be established. Self-reported data were gathered from participants, which may be subject to potential response bias. Given the data were collected using the online platform Qualtrics, findings may not represent non-Hispanic Black and Hispanic men with chronic conditions who have limited internet access or are unfamiliar with online surveys. No information was available about the type of chronic disease program the men were referred to or attended (e.g., evidence-based status, content included, format and structure, duration). The study did not examine providers’ knowledge about chronic disease programs in their local areas nor men’s barriers to program attendance, such as timing conflicts, work or family commitments, and geographical limitations, among others. Finally, this study only focused on individuals who had seen a health care provider in the past year. Consequently, the findings may not be generalizable to non-Hispanic Black and Hispanic men who lack regular access to health care.

## Conclusion

This study examined factors associated with adherence to health care provider referrals to attend disease prevention and management programs for non-Hispanic Black and Hispanic men with chronic conditions. While provider referrals to chronic disease programs were limited, those who received referrals were more likely to attend chronic disease programs, emphasizing the effectiveness of provider recommendations and underscoring the critical role of providers in guiding patients toward effective interventions. To amplify the impact of these referrals, additional efforts may be needed to raise awareness about chronic disease programs among providers and their patients, which could result in increased referrals and program attendance, and the delivery of more comprehensive support for non-Hispanic Black and Hispanic men with chronic conditions.
